# Supplementary material for: Conditional chemoconnectomics (cCCTomics) as a strategy for efficient and conditional targeting of chemical transmission
Source: eLife. 2024 Apr 30;12:RP91927. doi: 10.7554/eLife.91927 (PMC11060718; doi:10.7554/eLife.91927)
Supplement: Figure 3—source data 3. [file elife-91927-fig3-data3.zip › Figure 3 source data3 part 1.zip!/Figure 3-Source data 3-part1/1063f3-1-CRISPResso_Report_kboyr13q.zip!/CRISPResso_on_1063f3_3/9.Alleles_frequency_table_around_sgRNA_aatcacaccagtgggagcac.pdf]

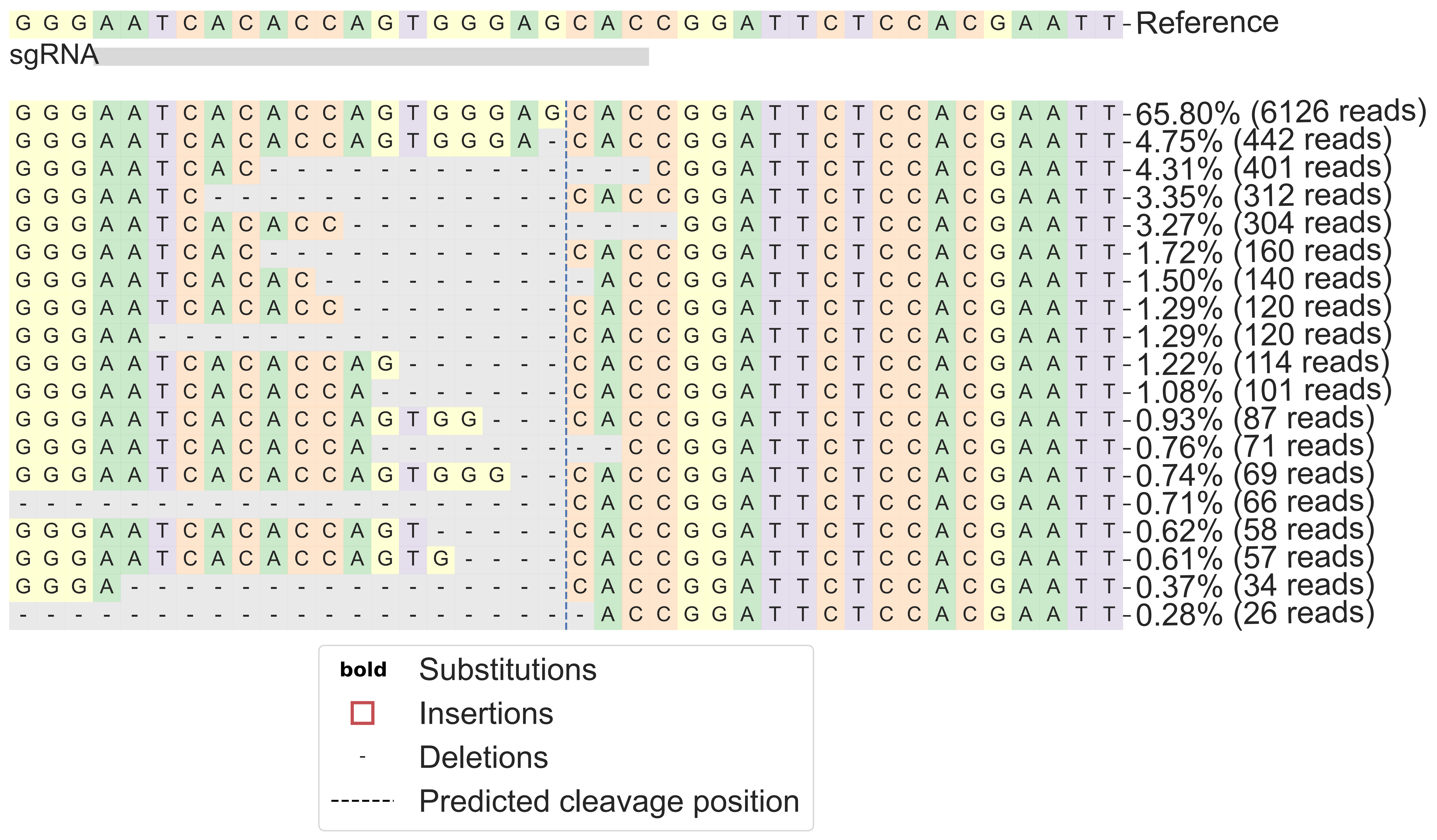
GGGAATCACACCAGTGGGAGCACCGGATTCTCCACGAATT Reference
sgRNA
GGGAATCACACCAGTGGGAGCACCGGATTCTCCACGAATT 65.80% (6126 reads)
GGGAATCACACCAGTGGGA-CACCGGATTCTCCACGAATT 4.75% (442 reads)
GGGAATCAC--------------CGGATTCTCCACGAATT 4.31% (401 reads)
GGGAATC-------------CACCGGATTCTCCACGAATT 3.35% (312 reads)
GGGAATCACACC------------GGATTCTCCACGAATT 3.27% (304 reads)
GGGAATCAC-----------CACCGGATTCTCCACGAATT 1.72% (160 reads)
GGGAATCACAC----------ACCGGATTCTCCACGAATT 1.50% (140 reads)
GGGAATCACACC--------CACCGGATTCTCCACGAATT 1.29% (120 reads)
GGGAA---------------CACCGGATTCTCCACGAATT 1.29% (120 reads)
GGGAATCACACCAG------CACCGGATTCTCCACGAATT 1.22% (114 reads)
GGGAATCACACCA-------CACCGGATTCTCCACGAATT 1.08% (101 reads)
GGGAATCACACCAGTGG---CACCGGATTCTCCACGAATT 0.93% (87 reads)
GGGAATCACACCA---------CCGGATTCTCCACGAATT 0.76% (71 reads)
GGGAATCACACCAGTGGG--CACCGGATTCTCCACGAATT 0.74% (69 reads)
--------------------CACCGGATTCTCCACGAATT 0.71% (66 reads)
GGGAATCACACCAGT-----CACCGGATTCTCCACGAATT 0.62% (58 reads)
GGGAATCACACCAGTG----CACCGGATTCTCCACGAATT 0.61% (57 reads)
GGGA----------------CACCGGATTCTCCACGAATT 0.37% (34 reads)
---------------------ACCGGATTCTCCACGAATT 0.28% (26 reads)
bold Substitutions
Insertions
Deletions
Predicted cleavage position
